# Risk Factors for Chronic Mastitis in Morocco and Egypt

**DOI:** 10.1155/2013/184921

**Published:** 2013-11-14

**Authors:** Hanna N. Oltean, Amr S. Soliman, Omar S. Omar, Tamer F. Youssef, Mehdi Karkouri, Azza Abdel-Aziz, Ahmad Hablas, Taylor Blachley, Ali Tahri, Sofia D. Merajver

**Affiliations:** ^1^University of Michigan School of Public Health, Ann Arbor, MI 48109-2029, USA; ^2^Department of Epidemiology, University of Nebraska Medical Center College of Public Health, Omaha, NE 68198-4395, USA; ^3^Cairo University Medical School, Cairo 11796, Egypt; ^4^Mansoura University Hospital, Mansoura, Dakahlia 35516, Egypt; ^5^Hassan II University, Ibn Rochd Hospital, Casablanca 77110, Morocco; ^6^Tanta Cancer Center, Dr. Nagati Street, Tanta 31512, Egypt; ^7^L'Hopital Ibn Tofail, 40000 Marrakech, Morocco; ^8^University of Michigan Medical School, Ann Arbor,MI 48109-5948, USA

## Abstract

Chronic mastitis is a prolonged inflammatory breast disease, and little is known about its etiology. We identified 85 cases and 112 controls from 5 hospitals in Morocco and Egypt. Cases were women with chronic mastitis (including periductal, lobular, granulomatous, lymphocytic, and duct ectasia with mastitis). Controls had benign breast disease, including fibroadenoma, benign phyllodes, and adenosis. Both groups were identified from histopathologically diagnosed patients from 2008 to 2011, frequency-matched on age. Patient interviews elicited demographic, reproductive, breastfeeding, and clinical histories. Cases had higher parity than controls (OR = 1.75, 1.62–1.90) and more reported history of contraception use (OR = 2.73, 2.07–3.61). Cases were less likely to report wearing a bra (OR = 0.56, 0.47–0.67) and less often used both breasts for breastfeeding (OR = 4.40, 3.39–5.72). Chronic mastitis cases were significantly less likely to be employed outside home (OR = 0.71, 0.60–0.84) and more likely to report mice in their households (OR = 1.63, 1.36–1.97). This is the largest case-control study reported to date on risk factors for chronic mastitis. Our study highlights distinct reproductive risk factors for the disease. Future studies should further explore these factors and the possible immunological and susceptibility predisposing conditions.

## 1. Introduction

Chronic mastitis (CM) is a group of diseases characterized by chronic inflammation of the breast, affecting mainly women of reproductive age in their fourth decade [1–3]. CM is histopathologically defined as inflammation of the breast, with the microabscess formation and/or the presence of granulomas [1]. This disease generally involves the breast unilaterally and may affect every quadrant region except for the subareolar area [2]. Cases mainly present with a breast mass, which may involve the overlying skin or penetrate the underlying pectoralis muscle with nipple retraction, sinus formation, and axillary lymphadenopathy [1]. Other symptoms may include galactorrhea, inflammation, pain, peau d'orange, tumorous indurations, nipple retraction and/or discharge, diffuse heaviness and enlargement, and ulcerations of the skin [4]. The disease may be locally aggressive with a recurrence rate between 16% and 50% [1]. Due to this variable clinical presentation and these similarities in symptoms as well as clinical and radiological findings with inflammatory breast cancer, diagnosis is difficult and must be confirmed histopathologically after surgical excision or core biopsy [1]. Diagnosis of CM should be done after exclusion of other causes of infective lesions of the breast including brucellosis, filariasis, actinomycosis, sarcoidosis, histoplasmosis, Wegner's granulomatosis, giant-cell arteritis, duct ectasia, fat necrosis, and breast cancer [2, 4]. 

Known etiologies of CM are diverse and include diabetes, lupus erythematosus, *Mycobacterium tuberculosis*, *Staphylococcus aureus*, and *Candida albicans, *as well as several species of *Corynebacterium *and other rare syndromes and infections [3–5]. Several other suspected predisposing or related diseases have been mentioned in the literature, including Weber-Christian disease, Sjogren's syndrome, hyperprolactinemia, IgG4 sclerosing disease, immune response to local trauma, and even cat scratch disease [6–10]. However, many cases of CM are idiopathic, presenting difficulties for determining the etiology and optimal treatment. 

Nearly all published case series on CM find that the average age of women at presentation is in their fourth decade [1–4, 11–14]. Studies have suggested risk factors involving parity, breastfeeding habits, contraception use, socioeconomic status, and treatment patterns; however, all of these stated risks are based on physicians' observations in small case series reports and have not been studied epidemiologically. 

The prevalence of CM is generally reported at less than 1% worldwide among women presenting with breast problems in studied hospitals; however, reports vary greatly by country and ethnic group. Reports from Pakistan show prevalence at less than 1% among women undergoing biopsy for breast diseases [15], and a study of granulomatous mastitis (GM) in the United States demonstrated a prevalence of less than 1% among women who underwent biopsy for breast diseases [11]. In the UK, periductal mastitis was found in 0.98% of patients presenting at the breast unit [16]. A second US-based study of GM found a prevalence of 2.4/100,000 women; however, the prevalence was 12 times higher among Hispanic women [17]. A study in Saudi Arabia found idiopathic granulomatous mastitis (iGM), one form of chronic mastitis, to be 1.8% of cases presenting with breast diseases [12]; likewise, a study in Turkey found that 6.8% of patients undergoing surgery for benign breast diseases had GM [18]. Initial reports from hospitals in Morocco [19] and Egypt [20], as well as our review of chronic mastitis diagnoses at the Department of Pathology at the National Cancer Institute of Cairo University, indicate that the disease may be less rare than in the developed countries, with prevalence rates estimated between 1% and 10%. However, due to the lack of access to specialized care, cases captured in tertiary hospitals and cancer centers are likely to be a very low estimate of total prevalence within these countries. 

Given the rarity of CM worldwide, little is known about etiology, risk factors, and treatment. A thorough literature review of PubMed, Scopus, and ISI Web of Knowledge using the search term “breast disease OR mastitis OR chronic mastitis OR granulomatous mastitis” and covering 1960 through 2011 revealed that almost all published studies are hospital-based case-series reports. The largest series we identified involved 54 patients with CM [11]. The only exception is one small retrospective case-control study of 18 cases of GM [14]. Therefore, a major gap seems to exist in the literature regarding epidemiological study of chronic mastitis. 

The current state of knowledge on CM is inadequate to inform treatment protocols, prevention efforts, or patient education. No risk factors are statistically shown to be associated with CM, severely limiting the potential prevention efforts. The lack of information, combined with the relatively high estimated prevalence in North Africa, created an optimal setting for the study of a neglected rare disease. The aim of this study was to identify potential risk factors for CM to inform future clinical research and possible prevention interventions. Based on a previous case-series work, we hypothesized that breastfeeding and reproductive factors would have important associations with diagnosis of CM.

## 2. Materials and Methods

### 2.1. Study Population

We preformed a retrospective hospital-based case-control study and identified 85 cases of CM and 112 controls from 5 hospitals in Morocco and Egypt. The hospitals in Morocco included L'Hopital Ibn Rochd, Hassan II University, in Casablanca, and L'Hopital Ibn Tofail in Marrakech. The hospitals in Egypt were Cairo University Medical School in Cairo, the Mansoura University Oncology Center in Mansoura city (in the East Nile Delta Region), and the Tanta Cancer Center in Tanta, in the center of the Nile Delta Region. These hospitals were chosen because of their relatively high reported number of CM cases. Cases were defined as any female patient with histopathological diagnosis of chronic mastitis (periductal, lobular, granulomatous, lymphocytic, and duct ectasia with mastitis) seen at the study hospitals between 2008 and 2011. The only exclusion criterion was previous diagnosis of malignancy. 

Controls were women with histopathologic diagnoses of other forms of benign breast diseases, excluding mastitis and malignancy, and including fibroadenoma, benign phyllodes, and adenosis, diagnosed at the same hospitals during the same period. The control conditions were chosen based on no suspected association with breast cancer. Cases and controls identified from pathology records of each collaborating hospital were frequency age-matched within ±5 years. Pathological records of the study subjects were linked to their medical records, and both were abstracted for relevant information. Any contact information available from medical records was used to contact and obtain consent from patients, as well as to conduct a follow-up interview. The consent process and interview were generally conducted in tandem by an Arabic-speaking interviewer over the telephone. At the Mansoura study site in Egypt, interviews were conducted in a face-to-face format at the hospital. No incentive was provided to interview, and the overall response rate among those contacted was 89%.

**Figure 1:**
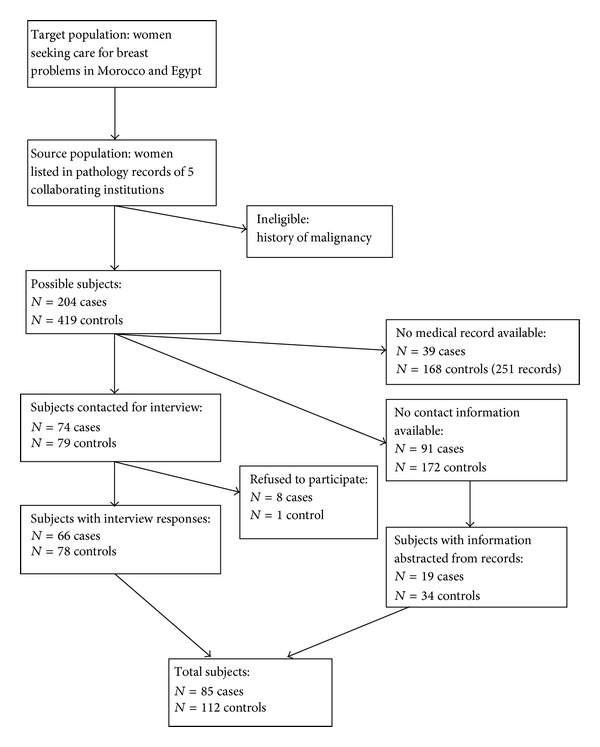
Selection of study participants.

The 2008–2011 databases of the pathology departments of the participating hospitals included 204 cases and 419 controls meeting the study criteria (Figure 1). Of these subjects originally identified, 165 cases and 251 controls were linked to existing medical records. Seventy-four cases and 79 controls had working contact information and were contacted for interviews. Of these subjects, 66 cases and 78 controls gave interview responses. An additional 19 cases and 34 controls had sufficient information contained in their records to complete the interview questions and were included in the analysis, for a total of 85 cases and 112 controls.

### 2.2. Data Collection

Interviewer-administered questionnaire included questions on demographics, reproductive history, breastfeeding history, hormone use, menstrual and menopause history, and occupational history, as well as a section on description of symptoms and treatment for their breast diseases. This questionnaire was a shortened version of a risk-factor assessment that was pilot-tested for reliability and validity in Morocco and Egypt [21] and is currently in use in our ongoing study of the epidemiology of breast cancer in North Africa. The study questionnaire was translated from English into Arabic by an experienced native-speaking translator. The study protocol was approved by the Institutional Review Board at the University of Michigan, as well as by ethics boards at all collaborating institutions.

### 2.3. Statistical Analysis

Data were compiled and imported into SAS, version 9.1, statistical software (SAS Institute, Car, NC, USA) for statistical analysis. Crude associations were assessed by frequency tables and descriptive statistics, and differences between cases and controls were tested by Chi-square tests or Fisher's exact tests for categorical variables and by Student's *t*-tests for continuous variables, with a two-sided significance level of *α* = 0.05. Bivariate analysis was performed using logistic regression, adjusted for age and hospital site in the model to control matching factors, to assess significance of variables as risk factors for case status. Due to the small sample size and the large number of strata created in the presence of two matching factors, conditional logistic regression was not used. 

The dataset was then imputed twenty times to allow for complete-case analysis by logistic regression. Imputation was carried out using IVEware, Imputation and Variance Estimation Version 0.2, Survey Methodology Program (Survey Research Center, Institute for Social Research, University of MI, USA). Following imputation, the complete set of twenty datasets was used to conduct bivariate analyses and multivariate analyses, adjusted for age and study site, to model predictors of the dichotomous outcome cases status and create an integrated predictive model. In the crude modeling of variables, 10 factors were shown to be significant at alpha ≤ 0.05. These variables were then included in an adjusted model, and all but 1 variable remained significant. This variable was “breastfed at least one child,” as its significance was accounted for in the variables “breastfed all children” and “did not breastfeed.” Additionally, 3 interaction terms were shown to significantly predict case status in the fully adjusted model, and these were included in the final analysis.

## 3. Results

Table 1 provides information on the sociodemographic characteristics of the study population, divided by case status and compared by Chi-square or Fischer's exact tests. Nineteen cases were recruited from Morocco and 66 cases from Egypt. The median age of cases at presentation was 33 (range: 17–59 years). Cases and controls had similar rates of urban versus rural residence (63% and 61%, resp.); however, controls were significantly more likely to be employed outside home (32% of controls versus 11% of cases, *P* = 0.005). 

Table 2 compares reproductive characteristics between cases and controls. Significantly larger numbers of cases (96%) were parous, compared to only 67% of controls (*P* < 0.0001). Additionally, cases had a higher average parity of 3.46 births than controls, 2.30 births (*P* = 0.0002). Median ages at first birth (22 years) and last birth (29 years) were the same for cases and controls. Similar frequencies of miscarriage (12%), irregular menstruation (18%), and infertility (13%) were seen among cases and controls. A significantly higher proportion of cases reported past use of contraceptive methods (*P* = 0.005), but when broken down by type of contraception used, no difference was seen. For those for whom information was available, 76% of cases were pre-menopausal; the distribution between menopausal statuses did not differ significantly for cases and controls. However, average age at menopause was 1.25 years lower for cases than for controls.

Table 3 shows information on breastfeeding characteristics of the study population. Among parous women, no significant differences were seen between cases and controls regarding the number of those who breastfed all of their children or who breastfed at least one child. However, significant differences were seen in the history of always using both breasts in feeding, with significantly lower rates among cases (51%) than controls (83%); *P* = 0.0019. Cases were less likely to wear a bra (78%) than controls (90%); *P* = 0.071.

Table 4 depicts information on the characteristics of the breast problems experienced by subjects in the study. Average age at breast problem was 35.44 for cases and 35.70 for controls. Cases were more likely to experience their breast problem in the left breast (55%) than the right breast (34%); however, this information was reported for too few of the cases for the finding to be significant. The majority of the cases were granulomatous mastitis (*N* = 37, 43.5%), one of the types of chronic mastitis included. In terms of temporal proximity to lactation (defined as problem within 6 months of lactation), no significant differences were seen between cases and controls; however, information was missing for the majority of both cases and controls. The main symptom of the breast problem was most likely to be swelling for both cases (40.0%) and controls (74.6%), but cases were more likely to report abscess, secretions, and redness. 

Treatment administered to cases was most likely to be antibiotics or radiation; controls reported other treatments or radiation as most common. Too little clinical information was reported for these differences to be significant.

Results from the adjusted bivariate analysis and fully adjusted logistic regression model, completing postimputation, are shown in Table 5. The bivariate analysis found 10 variables to be significant: 9 of these were included in the fully adjusted model after stepwise addition. In the multivariate analysis, several potentially important predictors were found to be significantly associated with an outcome of CM. Parity was significantly associated with an outcome of CM; cases experienced a 75% increase in odds of each additional birth. Cases were more likely to have a history of contraception use (OR = 2.73, 2.07–3.61), but this significance could not be related to type of contraception. Postmenopausal status was found to be protective against chronic mastitis when compared to premenopausal status (OR = 0.62, 0.49–0.79). 

A case of CM was 3.76 times more likely to have breastfed all of her children than a control, and cases who breastfed were 4.40 times more likely to have not alternated breasts when breastfeeding. Cases wore a bra less often than controls (OR = 0.56, 0.47–0.67). Temporal proximity of problem to pregnancy and lactation was also found to be significant in the final model, with cases being 67% more likely than controls to have experienced their breast problem during pregnancy, lactation, or within 6 months after lactation. A woman with CM was found to have 0.71 times lower odds of maintaining employment outside home than a control subject. Additionally, variables associated with reporting mice in the study subject's neighborhood or household were found to be risk factors; cases had a 47% increase in odds of reporting a campaign to control mice in their neighborhoods and a 63% increase in odds of reporting attempts to control mice in their households.

Interactions between parity and contraception were found to be significant; likewise, the effect of whether or not a woman breastfed all of her children varied across levels of parity. Both effects were less than additive (OR = 0.86, 0.80–0.93, and OR = 0.80, 0.73–0.87, resp.), with the effect of breastfeeding all children decreasing with each additional birth. Use of only one breast as a risk factor appeared to have a significant interaction with whether or not the breast problem was associated with lactation (OR = 0.32, 0.22–0.46). 

## 4. Discussion

Previous studies have suggested risk factors for CM involving parity, breastfeeding habits, contraception use, socioeconomic status, and treatment patterns; however, ours is the first study to present statistically significant findings [3, 17, 22]. Our results indicate that the strongest measured risk factors for CM are breastfeeding all children and not alternating breasts when breastfeeding. These risks match observations made by clinicians and reported in published case-series [3, 4, 17]. Several additional factors in this study appear to be associated with chronic mastitis, including premenopausal status, employment, higher parity, use of contraceptives, not wearing a bra, and reporting mice in one's household. Some of these coincide with factors that have been previously identified as potential risk factors, and all can potentially lead to hypothesis-based testing for mechanisms of etiology. 

History of contraception has been suggested in previous studies as a potential risk factor [4, 7, 11, 22]. In our study, any history of contraceptive use was found to be statistically more common in cases than controls, adding evidence to the importance of contraceptive use as a risk factor for CM. However, individual types of contraceptives could not be associated with case outcome, indicating that further research should be done to establish risk behavior.

**Figure 2:**
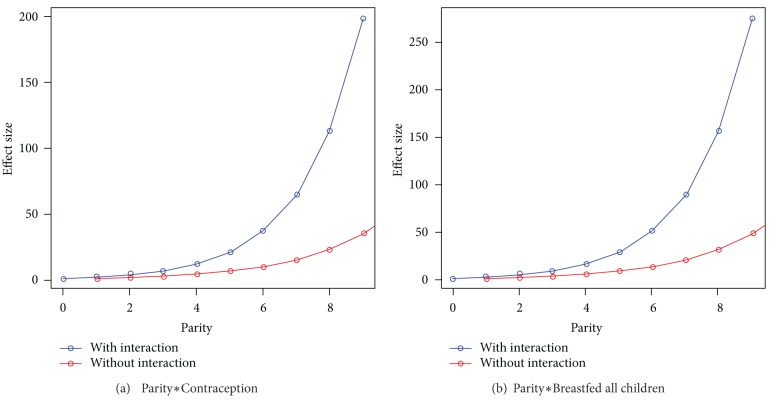
Graphical depiction of interaction terms Parity∗Contraception and Parity∗Breastfed all children. (a) Interaction of parity by contraception and (b) interaction of parity by breastfed all children. In both figures, blue line is in the presence of the interaction term, and red line is in the absence of the interaction term.

Suspected associations with pregnancy have been reported previously in case-series [3, 4, 7, 11, 14, 17]. Even after controlling for breastfeeding, parity was associated with CM in our study, indicating that it has a statistical significance outside its relation to breastfeeding. Interactions between parity and any history of contraception were found to be significant, indicating that the effect of history of contraception use varied across levels of parity. Specifically, the effects of parity and contraception were less than additive (OR = 0.86). This indicates that there is only so much explanatory power that can be attributed to contraception with each increasing birth—for each additional child that a woman has, the added importance of history of contraception use decreases. This concept of less-than-additive interaction is illustrated in Figure 2(a).

Breastfeeding has previously been correlated with CM in several case-series [4, 7, 17, 22]; however, in another study, no association was found [13]. In our study, breastfeeding all children was found to be very significant in the full model, accounting for the significance of breastfeeding at least one child. This finding is probably related to the fact that cases were more likely than controls to experience their breast problem during or within 6 months after lactation, although it was independently significant. However, this strong effect of breastfeeding all children decreases with increasing parity, as the interaction between parity and breastfeeding all children is less than additive (OR = 0.80, Figure 2(b)). The association of breastfeeding all children is seen to decrease with each additional birth, tempering the effect of breastfeeding all children in the presence of high parity. Further research is needed to understand the mechanism of this effect.

One of the main expected risk factors, as communicated by clinicians and reported in the literature, is the failure to alternate between breasts when breastfeeding [12]. This practice was found to be highly significant as a predictor of CM in our study. However, among those with a problem during lactation, the importance of using both breasts or not breastfeeding at all decreases in explaining case status. This interaction indicates that the present study cannot separate cause and effect—whether a woman experiences a problem because she is breastfeeding from only one breast or whether she breastfeeds in this pattern due to a breast problem. This problem has been described elsewhere, in the use of mastitis-related terms such as “milk stasis,” “retention,” “obstruction,” or “breast engorgement,” since these processes are considered as both predisposing factors and direct consequences of the disease [23]. 

Nearly all published reports [1–4, 11–14] on CM find that the average age of women at presentation is in their fourth decade. Our study likewise found the average age at presentation to be 35 years. Additionally, menopausal status was also found to be significant in the final model, with postmenopausal status seen to be protective. This finding correlates with the view of CM as a reproductive-age problem often associated with pregnancy and lactation. Other protective factors seen in this study include being employed outside home and wearing a bra, both of which could potentially be related to higher socioeconomic status. These associations have not been previously reported in the literature.

We included the condition of having mice in one's household on the basis of a hypothesis relating mouse mammary tumor virus (MMTV) to breast cancer [24]. The results from the modeling of this variable indicate that there may be a dose-response relationship with the odds of case status increase. Therefore, exposure to mice may be related to an important risk factor for disease. However, the relationship to mice could also indicate lower socioeconomic status of cases, which is unlikely.

While presentation and treatment were not included in the final predictive models, due to temporal occurrence after presentation with disease, differences in presentation and treatment between cases and controls were seen. The main symptom of the breast problem was most likely to be swelling for both cases and controls, but cases were more likely to report abscess, secretions, and redness. This fits within descriptions of CM in the literature. Treatment administered to cases was most likely to be antibiotics or radiation; controls reported other treatments or radiation as most common. From the published literature, the optimal treatment of CM is still unclear [18, 25]. Reported case-series have described treatment with antibiotics, steroids, abscess drainage, wide surgical resection, and even mastectomy, with variable results [2, 25–30]. There are currently insufficient data to make definitive recommendations, but conservative treatment with steroids and surgery only if necessary is generally agreed upon, following the exclusion of infection [1, 2, 11, 25]. Successful treatment with prednisolone, methotrexate, and azathioprine has been reported [13, 25–29]. Antibiotics should only be used in the treatment of CM if a bacterial infection is identified [1], despite the prevalent use of antibiotics seen in this study and reported elsewhere [6]. No information was available on the identification of infection before prescription of antibiotics. In the presence of abscess, drainage is the first choice of treatment [1]. 

Data and prior studies on CM are extremely limited; however, when compared with the previously published associations, our results reveal similar findings. The majority of published papers and case reports have come from the developing countries, which has been hypothesized to be due to lower prevalence of CM in the developed countries, overdiagnosis in the developing countries, or underdiagnosis of tuberculosis mastitis in the developing countries [3, 7]. Additionally, this differential in reporting could reflect true differences in risk factors worldwide. A preponderance of reports have been documented as coming from Mediterranean countries and Asia [12]. A study in the USA found that Hispanic women had 12 times the risk of CM as non-Hispanic women [17], and a study in the UK found that women with CM were significantly less likely to be Caucasian [14]. This indicates that the disease may be more common in minority or developing-country settings, an idea that correlates with our findings in Morocco and Egypt.

The study has a limitation. Among those for whom medical records were available, many did not have working contact information. However, response rates were high among those we were able to contact. Conclusions drawn from this study therefore require an assumption of nondifferential missingness patterns between cases and controls, along with an assumption that those who could be contacted were a representative sample of all cases and controls. 

The case definition used in this study was wider than that commonly used to study CM, as the vast majority of the published literature studies specific types of CM. Due to the rarity of the disease and the commonalities in presentation and treatment, as well as a general lack of knowledge, we felt that it was appropriate to study these conditions together. While recall bias is often a concern in case-control studies, the fact that both our case and control populations experienced past breast disease potentially mitigates this concern. There is still some temporal ambiguity; however, many of the potential risk factors surround pregnancy and lactation, and dates of pregnancy were known, eliminating some ambiguity. Despite these limitations, this study is a landmark picture of the epidemiology of CM. Clinical observations long reported in the literature but never studied broadly now have statistical significance, opening the way for future studies. 

Chronic mastitis is a rare inflammatory disease of the breast. Little is known about the etiology or risk factors for the disease. Because studies on risks, etiologies, and treatments are lacking, women experiencing CM are often incorrectly treated. Despite its relatively severe presentation and lack of standard treatment protocol, we are unaware of any controlled analytic studies modeling risk factors for CM to date. Therefore, we believe that we have conducted the largest case-control study of CM in the literature, investigating the epidemiology and potential risk factors for the disease. Our findings indicate that the disease typically occurs in the fourth decade of life and has a statistically significant association with pregnancy/parity, lactation, breastfeeding patterns, menopausal status, employment status, history of use of contraception, having mice in one's household, and not wearing a bra. Particularly strong associations were found between CM and breastfeeding patterns and CM and parity. These data provide the first comprehensive statistical analysis of potential risk factors for CM. Further studies are required to parse out understandings of mechanisms, establish temporality, and further understand risks and etiologies of chronic mastitis.

## Figures and Tables

**Table 1:** Demographic characteristics of 197 women who participated in a case-control study of chronic mastitis, Morocco and Egypt, 2011. Unadjusted, pre-imputation.

Characteristics^(cases,controls)^	Case		Control		*P* ^a^
	*N*	%	*N*	%	
Total^(85,112)^	85	100	112	100	
Age^(83,112)^					0.035^b∗^
≤19	1	1.20	6	5.41	
20–29	13	15.66	32	28.57	
30–39	32	38.55	22	19.64	
40–49	20	24.10	28	25.00	
50–59	11	13.25	16	14.29	
≥60	6	7.23	7	6.25	
Country^(85,112)^					0.7732
Morocco	19	22.35	27	24.11	
Egypt	66	77.65	85	75.89	
Residence^(79,95)^					0.7619
Urban	50	63.29	58	61.05	
Rural	29	36.71	37	38.95	
Employment^(55,71)^					0.0045*
Housewife	49	89.09	48	67.61	
Other	6	10.91	23	32.39	
Mice in neighborhood^(44,75)^					0.1005
Yes	9	20.45	26	34.67	
No	35	79.55	49	65.33	
Campaign to control mice in neighborhood^(17,36)^					0.1581
Yes	5	29.41	18	50.00	
No	12	70.59	18	50.00	
Control mice in household^(36,56)^					0.0205*
Yes	14	38.89	10	17.86	
No	22	61.11	46	82.14	

^a^ *P* value from chi-square test.

^b^ *P* value from Fisher's Exact test.

*Significant at *α* ≤ 0.05.

**Table 2:** Reproductive characteristics of 197 women who participated in a case-control study of chronic mastitis, Morocco and Egypt, 2011.

Characteristics	Case		Control		*P* ^a^
	*N*	%	*N*	%	
Total^(85,112)^	85	100	112	100	
Currently pregnant^(56,76)^					0.722^b^
Yes	4	7.14	4	5.26	
No	52	92.86	72	94.74	
Parity^(81,98)^					<.0001^b∗^
0	1	1.23	28	28.57	
1-2	26	32.10	24	24.49	
3-4	36	44.44	34	34.69	
≥5	18	22.22	12	12.24	
If parous, age at first birth^(51,51)^					0.0219^b∗^
≤18	5	9.80	13	25.49	
19–24	33	64.71	18	35.29	
25–30	10	19.61	16	31.37	
≥31	3	5.88	4	7.84	
If parous, age at last birth^(51,51)^					0.602^b^
≤18	0	0	2	3.92	
19–24	7	13.73	9	17.65	
25–30	22	43.14	19	37.25	
≥31	22	43.14	21	41.18	
Previous miscarriage^(52,66)^					0.734
Yes	6	11.54	9	13.64	
No	46	88.46	57	86.36	
Ever infertile^(63,77)^					0.959
Yes	8	12.70	10	12.99	
No	55	87.30	67	87.01	
History of contraception^(79,97)^					0.0053
Yes	54	68.35	46	47.42	
No	25	31.65	51	52.58	
Type of contraception^(41,37)^					0.528^b^
Pill	18	43.90	18	48.65	
Shot	4	9.76	5	13.51	
Patch	2	4.88	4	10.81	
Implant (IUD)	17	41.46	10	27.03	
Age started contraception^(35,30)^					0.857^b^
≤19	4	11.43	6	20.00	
20–24	13	37.14	10	33.33	
25–29	10	28.57	8	26.67	
≥30	8	22.86	6	20.00	
Current menstrual cycle^(70,91)^					0.542
Yes	53	75.71	65	71.43	
No	17	24.29	26	28.57	
Regular menstrual cycle^(65,91)^					0.748
Yes	53	81.54	76	83.52	
No	12	18.46	15	16.48	
Menopausal status^(80,102)^					0.727
Pre-	61	76.25	80	78.43	
Post-	19	23.75	22	21.57	
Age at menopause^(15,19)^					0.580^b^
≤45	4	26.67	2	10.53	
46–50	4	26.67	6	31.58	
>50	7	46.67	11	57.89	

^a^ *P* value from Chi-square test.

^b^ *P* value from Fisher's exact test.

*Significant at *α* ≤ 0.05.

**Table 3:** Breastfeeding characteristics of 197 women who participated in a case-control study of chronic mastitis, Morocco and Egypt, 2011.

Characteristics	Case		Control		*P* ^a^
	*N*	%	*N*	%	
Total^(85,112)a^	85	100	112	100	
If parous, breastfed all children^(52,49)^					0.690
Yes	43	82.69	39	79.59	
No	9	17.31	10	20.41	
If parous, breastfed at least one child^(67,66)^					0.365^b^
Yes	63	94.03	59	89.39	
No	4	5.97	7	10.61	
If history of breastfeeding, always used both breasts^(49,40)^					0.0019*
Yes	25	51.02	33	82.50	
No	24	48.98	7	17.50	
Wears a bra^(55,77)^					0.071
Yes	43	78.18	69	89.61	
No	12	21.82	8	10.39	

^a^ *P* value from Chi-square test.

^b^ *P* value from Fisher's exact test.

*Significant at *α* ≤ 0.05.

**Table 4:** Characteristics of breast problems of 197 women who participated in a case-control study of chronic mastitis, Morocco and Egypt, 2011.

Characteristics	Case		Control		*P* ^a^
	*N*	%	*N*	%	
Total^(85,112)a^	85	100	112	100	
Side of breast problem^(38,52)^					0.659^b^
Right	13	34.21	23	44.23	
Left	21	55.26	25	48.08	
Both	4	10.53	4	7.69	
Problem within 6 months of lactation^(39,54)^					0.311^b^
Yes	6	15.38	4	7.41	
No	33	84.62	50	92.59	
Main symptom of breast problem^(40,54)^					<0.0001^∗b^
Abscess	9	22.5	1	1.82	
Secretions	5	12.5	3	5.45	
Redness	9	22.5	1	1.82	
Swelling	16	40.0	41	74.55	
Pain	1	2.5	8	14.55	
Other	0	0	1	1.82	
Treatment of problem^(38,52)^					<0.0001^∗b^
Antibiotics	25	62.5	11	20.37	
Pain medication	2	5.0	4	7.41	
Hot compress	0	0	1	1.85	
Abscess drainage	1	2.5	0	0	
Radiation	9	22.5	13	24.07	
Other	3	7.5	20	37.04	
Not treated	0	0	5	9.26	

^a^ *P* value from Chi-square test.

^b^ *P* value from Fisher's exact test.

*Significant at *α* ≤ 0.05.

**Table 5:** Conditional logistic regression for predictors of chronic mastitis. Model 1: adjusted only for age and study site and Model 2: adjusted for all significant variables, interactions (*n* = 197).

Predictors	Model 1		Model 2	
	OR	95% CI	OR	95% CI
Employment				
Housekeeper/unemployed	1.00	ref	1.00	ref
Other	0.53*	(0.46, 0.61)	0.71*	(0.60, 0.84)
Mice				
No mice reported	1.00	ref	1.00	ref
Mice in neighborhood	0.97	(0.80, 1.16)	0.91	(0.73, 1.13)
Campaign to control mice in neighborhood	1.60*	(1.36, 1.87)	1.47*	(1.10, 1.98)
Control mice in household	1.19	(0.92, 1.52)	1.63*	(1.36, 1.97)
Parity	1.62*	(1.54, 1.70)	1.75*	(1.62, 1.90)
Age at first birth	0.99	(0.98, 1.01)	—	—
History of contraception				
No	1.00	ref	1.00	ref
Yes	2.80*	(2.42, 3.24)	2.73*	(2.07, 3.61)
Menopausal status				
Pre-	1.00	ref	1.00	ref
Post-	0.66*	(0.54, 0.81)	0.62*	(0.49, 0.79)
Age at menopause	1.02	(0.99, 1.05)	—	—
Breastfed all children				
No	1.00	ref	1.00	ref
Yes	3.04*	(2.64, 3.50)	3.76*	(2.69, 5.25)
Breastfed at least one child				
No	1.00	ref	—	—
Yes	4.89*	(4.11, 5.81)	—	—
Breastfed with both breasts				
Yes	1.00	ref	1.00	ref
No	2.75*	(2.32, 3.27)	4.40*	(3.39, 5.72)
Did not breastfeed	0.30*	(0.25, 0.36)	1.54*	(1.07, 2.21)
Wears a bra				
No	1.00	ref	1.00	ref
Yes	0.68*	(0.58, 0.79)	0.56*	(0.47, 0.67)
Proximity to pregnancy/lactation				
No proximity	1.00	ref	1.00	ref
Problem during pregnancy, lactation, or within 6 months after lactation	1.20*	(1.04, 1.38)	1.67*	(1.32, 2.11)
Parity* contraception	—	—	0.86	(0.80, 0.93)
Parity* breastfed all	—	—	0.80	(0.73, 0.87)
Lactation problem*breastfed with both breasts				
Did not use both breasts	—	—	0.32	(0.22, 0.46)
Did not breastfeed	—	—	0.46	(0.31, 0.68)

*Significant at *α* ≤ 0.05.

## References

[1] Kok KY, Telisinghe PU (2010). Granulomatous mastitis: presentation, treatment and outcome in 43 patients. *The Surgeon*.

[2] Erozgen F, Ersoy YE, Akaydin M (2010). Corticosteroid treatment and timing of surgery in idiopathic granulomatous mastitis confusing with breast carcinoma. *Breast Cancer Research and Treatment*.

[3] Tuli R, O’Hara BJ, Hines J, Rosenberg AL (2007). Idiopathic granulomatous mastitis masquerading as carcinoma of the breast: a case report and review of the literature. *International Seminars in Surgical Oncology*.

[4] Diesing D, Axt-Fliedner R, Hornung D, Weiss JM, Diedrich K, Friedrich M (2004). Granulomatous mastitis. *Archives of Gynecology and Obstetrics*.

[5] Paviour S, Musaad S, Roberts S (2002). Corynebacterium species isolated from patients with mastitis. *Clinical Infectious Diseases*.

[6] Taniguchi Y, Kagawa T, Ishibashi A, Horino T, Kumon Y, Terada Y (2011). Weber-Christian disease associated with granulomatous mastitis: a variant type of Weber-Christian disease?. *Modern Rheumatology*.

[7] Akcan A, Akyildiz H, Deneme MA, Akgun H, Aritas Y (2006). Granulomatous lobular mastitis: a complex diagnostic and therapeutic problem. *World Journal of Surgery*.

[8] Ríos G, Peredo RA (2010). Lymphocytic mastitis preceding sjögren’s syndrome. *Puerto Rico Health Sciences Journal*.

[9] Gamblin TC, Nobles-James C, Bradley RA, Katner HP, Dale PS (2005). Cat scratch disease presenting as breast mastitis. *Canadian Journal of Surgery*.

[10] Cheuk W, Chan ACL, Lam W-L (2009). IgG4-related sclerosing mastitis: description of a new member of the igg4-related sclerosing diseases. *American Journal of Surgical Pathology*.

[11] Larsen LJH, Peyvandi B, Klipfel N, Grant E, Iyengar G (2009). Granulomatous lobular mastitis: imaging, diagnosis, and treatment. *American Journal of Roentgenology*.

[12] Baslaim MM, Khayat HA, Al-Amoudi SA (2007). Idiopathic granulomatous mastitis: a heterogeneous disease with variable clinical presentation. *World Journal of Surgery*.

[13] Azlina AF, Ariza Z, Arni T, Hisham AN (2003). Chronic granulomatous mastitis: diagnostic and therapeutic considerations. *World Journal of Surgery*.

[14] Al-Khaffaf B, Knox F, Bundred NJ (2008). Idiopathic granulomatous mastitis: a 25-year experience. *Journal of the American College of Surgeons*.

[15] Ahmed R, Sultan F (2006). Granulomatous mastitis: a review of 14 cases. *Journal of Ayub Medical College*.

[16] Dixon JM, Ravisekar O, Chetty U, Anderson TJ (1996). Periductal mastitis and duct ectasia: different conditions with different aetiologies. *British Journal of Surgery*.

[17] Centers for Disease Control and Prevention (CDC) (2009). Idiopathic granulomatous mastitis in Hispanic women—Indiana, 2006–2008. *Morbidity and Mortality Weekly Report*.

[18] Ocal K, Dag A, Turkmenoglu O, Kara T, Seyit H, Konca K (2010). Granulomatous mastitis: clinical, pathological features, and management. *Breast Journal*.

[19] Belaabidia B, Essadki O, El Mansouri A, Sqalli S (2002). Idiopathic granulomatous mastitis: report of eight cases and review of the literature. *Gynecologie Obstetrique Fertilite*.

[20] Mokhtar N, Gouda I, Adel I (2007). *Cancer Pathology Registry 2003-2004 and Time Trend Analysis*.

[21] Soliman AS, Banerjee M, Lo A-C (2009). High proportion of inflammatory breast cancer in the population-based cancer registry of gharbiah, Egypt. *Breast Journal*.

[22] Verfaillie G, Breucq C, Sacre R, Bourgain C, Lamote J (2006). Granulomatous lobular mastitis: a rare chronic inflammatory disease of the breast which can mimic breast carcinoma. *Acta Chirurgica Belgica*.

[23] Contreras GA, Rodríguez JM (2011). Mastitis: comparative etiology and epidemiology. *Journal of Mammary Gland Biology and Neoplasia*.

[24] Levine PH, Pogo BG-T, Klouj A (2004). Increasing evidence for a human breast carcinoma virus with geographic differences. *Cancer*.

[25] Sakurai K, Fujisaki S, Enomoto K, Amano S, Sugitani M (2011). Evaluation of follow-up strategies for corticosteroid therapy of idiopathic granulomatous mastitis. *Surgery Today*.

[26] Lai ECH, Chan WC, Ma TKF, Tang APY, Poon CSP, Leong HT (2005). The role of conservative treatment in idiopathic granulomatous mastitis. *Breast Journal*.

[27] Jorgensen MB, Nielsen DM (1992). Diagnosis and treatment of granulomatous mastitis. *American Journal of Medicine*.

[28] Kim J, Tymms KE, Buckingham JM (2003). Methotrexate in the management of granulomatous mastitis. *ANZ Journal of Surgery*.

[29] Raj N, MacMillan RD, Ellis IO, Deighton CM (2004). Rheumatologists and breasts: immunosuppressive therapy for granulomatous mastitis. *Rheumatology*.

[30] Hladik M, Schoeller T, Ensat F, Wechselberger G (2011). Idiopathic granulomatous mastitis: successful treatment by mastectomy and immediate breast reconstruction. *Journal of Plastic, Reconstructive and Aesthetic Surgery*.

